# Laparotomy-Induced Peripheral Inflammation Activates NR2B Receptors on the Brain Mast Cells and Results in Neuroinflammation in a Vagus Nerve-Dependent Manner

**DOI:** 10.3389/fncel.2022.771156

**Published:** 2022-02-10

**Authors:** Jing Yang, Hong-Quan Dong, Yan-Hu Liu, Mu-Huo Ji, Xun Zhang, Hong-Yu Dai, Zhao-Chu Sun, Lu Liu, Jian Zhou, Huan-Huan Sha, Yan-Ning Qian, Qing-Guo Li, Hao Yao, Na-Na Li

**Affiliations:** ^1^Department of Anesthesiology, The First Affiliated Hospital of Nanjing Medical University, Nanjing, China; ^2^Department of Anesthesiology, The Second Affiliated Hospital of Nanjing Medical University, Nanjing, China; ^3^Cardiovascular Center, The Second Affiliated Hospital of Nanjing Medical University, Nanjing, China

**Keywords:** vagus, neuroinflammation, brain mast cells, glutamate, NR2B

## Abstract

**Background**: The pathophysiological mechanisms underlying postoperative cognitive dysfunction (POCD) remain unclear over the years. Neuroinflammation caused by surgery has been recognized as an important element in the development of POCD. Many studies also suggest that the vagus nerve plays an important role in transmitting peripheral injury signals to the central nervous system (CNS) and the resultant neuroinflammation. Previously, we have demonstrated that brain mast cells (BMCs), as the “first responders”, play a vital role in neuroinflammation and POCD. However, how the vagus nerve communicates with BMCs in POCD has not yet been clarified.

**Methods**: In the current study, we highlighted the role of the vagus nerve as a conduction highway in surgery-induced neuroinflammation for the first time. In our model, we tested if mice underwent unilateral cervical vagotomy (VGX) had less neuroinflammation compared to the shams after laparotomy (LP) at an early stage. To further investigate the roles of mast cells and glutamate in the process, we employed Kit^W-sh^ mice and primary bone marrow-derived MCs to verify the glutamate-NR2B axis on MCs once again.

**Results**: Our results demonstrated that there were higher levels of glutamate and BMCs activation as early as 4 h after LP. Meanwhile, vagotomy could partially block the increases and reduce neuroinflammation caused by peripheral inflammation during the acute phase. Excitingly, inhibition of NR2B receptor and knockout of mast cells can attenuateneuroinflammation induced by glutamate.

**Conclusion**: Taken together, our findings indicate that the vagus is a high-speed pathway in the transmission of peripheral inflammation to the CNS. Activation of BMCs triggered a neuroinflammatory cascade. Inhibition of NR2B receptor on BMCs can reduce glutamate-induced BMCs activation, neuroinflammation, and memory impairment, suggesting a novel treatment strategy for POCD.

## Introduction

Postoperative cognitive dysfunction (POCD) is characterized by a decline in cognitive performance, memory, and learning following surgery, particularly in aged patients (Yan et al., 2012). Previous studies have shown that the incidence of POCD in aged patients undergoing non-cardiac surgery was 25.8% and 9.9% at 1 week and 3 months respectively. In patients undergoing cardiac surgery, the incidence of POCD was as high as 39% at 3 months (Steinmetz et al., 2009; Kok et al., 2014). Therefore, understanding the pathophysiology of surgery-induced POCD is crucial in developing new pharmaceutic methods to alleviate symptoms. Multiple changes were found to regulate POCD, including neuroinflammation, oxidative stress, and neurotransmitter imbalances (Han et al., 2020). Neuroinflammation plays a key role in POCD (He et al., 2012; Wang et al., 2013). However, little was known on how peripheral surgery caused neuroinflammation.

Until now, scarce studies have demonstrated how inflammation was transducted from the periphery to the central nervous system (CNS) in POCD. Vagal afferents make up approximately 80% of the vagus nerve, with the central axonal terminal releasing glutamate as a main neurotransmitter (Chavan et al., 2017). A recent study has identified that the vagal afferents can sense local injury and transmit signals to CNS (Steinberg et al., 2016). Another study demonstrated that vagotomy prevented the neurobehavioral deficits induced by intestinal injection of pathological α-synuclein (Kim et al., 2019). These findings suggest that the vagus may be a key mediator in conducting the neuroinflammation signal. However, mechanisms underlying this process remain elusive.

In recent years, interactions between the nervous and immune systems have been reported to play important roles in regulating the pathology of POCD. Mast cell activity can be modulated by neurotransmitters, which allow them to control inflammation and immunity in the brain. Recently, Mast cells in the brain parenchyma (BPMCs) have been identified as the “first responder” to brain injury and as an initiator of the neuroinflammatory response (Skaper et al., 2017; Ocak et al., 2019). Mast cells (MCs) and neurons are closely aligned, both anatomically and functionally, throughout the body. For instance, in the thalamus, hypothalamus, and leptomeninges, BPMCs are adjacent to neurons and glial cells, and can activate them through cell-to-cell contact, as well as by releasing inflammatory and neurotoxic mediators (Theoharides et al., 2013). Moreover, our team found that BPMCs participate in surgery-induced cognitive impairment, inflammatory factor production, and blood-brain barrier (BBB) breakdown. We also found that BMCs regulate neuroinflammation and BBB dysfunction in the hypothalamus as an early activator (Zhang et al., 2016; Dong et al., 2019). In recent years, with the discovery of meningeal lymphatics, researchers have carried out many large single-cell sequencing and flow cytometry experiments on meninges, and found that there are also a large number of mast cells on meninges. Thus, we know that brain mast cells (BMCs) include not only brain parenchymal mast cells (BPMCs) but also meningeal mast cells (MMCs). However, the role of BPMCs and MMCs in neuroinflammation induced by peripheral inflammation has not been clarified.

The importance of glutamate abnormalities in mediating neurotransmitter imbalances in POCD has also been highlighted in recent studies (Han et al., 2020). As a primary excitatory neurotransmitter in the brain, glutamate plays crucial role in cognitive function as well as the development of neurodegenerative disorders (Culley et al., 2004; Li et al., 2015). Among the various subtypes of NMDA receptors, the NR2B subunit is closely related to epilepsy, learning, and memory impairment (Liu et al., 2012; Liu and Zhao, 2013). Mawhinney et al. (2012) revealed that spatial learning impairment corresponds to an acute and long-term increase in NR2B protein expression levels following surgery. In addition, Ghaffary et al. (2017) found that preoperative administration of NMDA antagonists protected patients from POCD and improved cognitive function 3 months after surgery. Several studies have also reported that MCs located within healing tendons express glutamate receptors, indicating that BMCs may respond to the increase of glutamate in neuroinflammation (Alim et al., 2021). However, mechanisms underlining the activation of BMCs by glutamate remain largely unknown.

In the current study, we explored the mechanisms of how peripheral inflammation was conducted into CNS. We hypothesized that peripheral inflammation leads to a vagus nerve-dependent activation of the NR2B on BMCs at an early stage and results in neuroinflammation and cognitive impairment.

## Methods

### Animals

Ninety-six male C57BL/6 wild-type (WT) mice (12 months old, 30–40 g) were obtained from the Model Animal Research Center of Nanjing University. Twenty-four C57BL/6-Kit^W-sh^/Kit^W-sh^ (Kit^W-sh^) mice (12 months old, 30–40 g) were generated in our laboratory, and colonies of these mice were maintained for our studies. All mice were housed on a 12 h:12 h light/dark cycle (lights on at 08:00) with free access to food and water. All procedures involving animals were performed according to the Guide for the Care and Use of Laboratory Animals of the National Institutes of Health and were approved by the Institutional Animal Care and Use Committee (IACUC) of Nanjing Medical University (No. 1911004).

### Laparotomy

Exploratory laparotomy (LP) was performed after the mice were anesthetized with 50 mg/kg pentobarbital sodium, which was administered intraperitoneally. Briefly, the abdominal region was shaved and sterilized, and a 1 cm midline incision was made to open the abdominal cavity. Then, we used sterile cotton swabs to explore the abdominal cavity. After that, the small intestine was exteriorized and left in the air for 20 min. The intestine was then returned to the abdominal cavity, and the wound was sutured in three layers (the peritoneal lining, abdominal muscle, and skin). Exterior wounds were dressed with antibiotic powder. To relieve incision pain in the animals, local anesthetics were used during incision closure. The incisions were then sutured, and 5% lidocaine ointment was applied to the wound.

### Cervical Vagotomy (VGX)

To prepare mice for cervical vagotomy (VGX) and avoid potential high mortality associated with bilateral cervical vagotomy, a unilateral cervical vagotomy was performed. Mice were anesthetized by i.p. administration of 50 mg/kg pentobarbital sodium, and the right cervical vagus nerve was exposed, ligated using 5–0 silk sutures, excised at least 1 cm, and divided from the internal jugular artery and vein. In sham-operated mice, the trunk of the vagus nerve was gently exposed and isolated from the surrounding tissue but not cut. All mice were permitted to recover for 2 h following the surgical procedure.

### Drug Administration by Stereotactic Injection

BPMCs are plentiful in the hypothalamus. Therefore, glutamate was injected into a central site in the ipsilateral hypothalamus to determine whether BPMCs are involved in glutamate-induced neuroinflammation. Mice were anesthetized by i.p. injection of pentobarbital sodium (50 mg/kg) and placed in the head frame of a stereotactic apparatus (Stoelting Instruments, USA) on a heating pad. Ophthalmic ointment was applied to the eyes prior to surgery. The scalp was shaved and scrubbed (with betadine and alcohol three times), and a, local anesthetic was applied (lidocaine 0.25%). The scalp was then incised through the midline. A guide cannula (Plastic One) was implanted into the right hypothalamus of each mouse (0.8 mm posterior to the bregma, 0.8 mm lateral to the midline and 4.6 mm ventral to the surface of the skull) at a 10° angle and secured to the skull using adhesive dental cement. The mice were observed during recovery and then returned to the animal facility and monitored daily. One week after the cannula implantation, the mice were separated into six groups according to the treatments they received: the saline group (0.9% saline, 1 μl), the Ro group (intrahypothalamic, i.h.t; 1 μM Ro25-6981/1 μl), the Glu-4 h group (i.h.t, 500 μM glutamate/1 μl), the Ro+Glu-4 h group (i.h.t., 1 μM Ro25-6981/1 μl+500 μM glutamate/1 μl), the Glu-24 h group (i.h.t., 500 μM glutamate/1 μl), and the Ro+Glu-24 h group (i.h.t, 1 μM Ro25-6981/1 μl + 500 μM glutamate/1 μl). After 30 min of Ro25-6981 injection, mice were injected with glutamate. Ro25-6981 (Ro) hydrochloride hydrate was purchased from Sigma Aldrich (Millipore Sigma, USA) and was a selective antagonist of the NR2B receptor. After drug administration, the mice were sacrificed, and their brains were collected for morphological and biochemical analyses.

### Generation and Culture of Bone Marrow-Derived MCs (BMMCs)

Femoral and tibial bones from the hind legs of C57BL/6 male mice were used for isolation of BMMCs. Briefly, femoral bone marrow and tibial bone marrow from each mouse were pooled together into one 15 ml centrifuge tube. Cells were placed in tissue culture flasks of appropriate size (e.g., T25 for cells from one mouse, T75 for cells from two mice). The bone marrow cells were grown in high-glucose DMEM (Gibco) supplemented with IL-3 (10 ng/ml, Sigma), SCF (10 ng/ml, Sigma), 10% heat-inactivated fetal bovine serum (FBS, Gibco), 1% penicillin-streptomycin (Gibco), and 2 mM L-glutamine (Gibco) at 37°C in a 5% CO_2_ incubator. The cells were maintained at a concentration of 0.5–1 × 10^6^ cells/ml with weekly medium changes. After a 4–6 week culture period, >85% of the cells in the culture were MCs, as determined by 0.1% toluidine blue staining and flow cytometry. BMMCs cultured over 5 weeks were used for subsequent experiments.

### Immunohistochemistry and Immunofluorescence

Briefly, after the experimental period, the mice were sacrificed, and the brain tissues were subjected to fixation using 4% paraformaldehyde (PFA) followed by embedding in paraffin. Then, we cut the brain tissue into thin slices (10 μm) according to the locations of the hippocampus and hypothalamus. After that, the tissue sections were first blocked with 1% BSA combined with 0.3% Triton X-100 and then washed in PBS (3×). The cells were first placed onto glass slides and fixed with PFA for 30 min. Then, the samples on slides were also blocked with 1% BSA combined with 0.3% Triton X-100 and washed in PBS (3×). After the first blocking step, the tissue sections/primary cells were incubated overnight (4°C in the dark) with the following primary antibodies. Single immunohistochemical staining for Iba-1 was performed using a rabbit polyclonal anti-Iba1 antibody (1:300, Wako). Mast cell immunofluorescence was labeled with avidin Alexa Fluor^TM^ 488 (1:250, Invitrogen) or mouse anti-tryptase (1:100, abcam), and NR2B protein was labeled with rabbit anti-NR2B (1:100, proteintech). All primary antibodies were diluted with PBS/1% BSA. Next, the tissue sections and/or primary cells were washed in PBS (3 × 5 min) and then incubated for 60 min with a fluorescent secondary antibody. For visualization of nuclei, DAPI (SouthernBiotech) staining was performed. Digital images were captured using a Leica fluorescence microscope. All photographs were taken at an original magnification of ×200, ×400, or ×630 with an oil objective. All 3D images were captured with z-stacks.

### MC Staining and Counting

Brain tissue sections and cell slides were prepared in advance, stained with 0.1% toluidine blue, and subjected to cell counting as previously described (Dong et al., 2019). The sections or slides were immersed in this staining solution for 30 min, washed twice with distilled water, and finally immersed in butyl acetate ester. The sections or slides were allowed to dry overnight. MCs were counted with Cell D software (Olympus) under double-blind conditions, and the counts are expressed as the number of cells per high-power visual field.

### Cytokine Assay

The levels of IL-6 and IL-1β were measured using ELISA kits (Invitrogen) according to the manufacturer’s instructions. Cellular supernatant was collected *via* centrifugation (300 × *g* for 10 min). Brain tissues were homogenized in ice-cold PBS, and the protein solution was obtained by centrifugation at 12,000× rpm for 15 min at 4°C. Assay diluent (50 μl) was added to each well, and then samples (50 μl) were added to each well. After incubation for 2 h at room temperature, each well was washed six times with wash buffer. After that, substrate solution was added to each well and incubated for 30 min without light. Then, a stop solution was added to each well. The optical density of each well was measured within 30 min using a microplate reader set to 450 nm.

### Glutamate Concentration Determination

The concentration of glutamate were measured using Glutamate Assay Kit (Jiancheng, China) according to the manufacturer’s instructions. Extract brain tissue protein according to the above method. Glutamate concentration is determined by an enzymatic assay, which results in a colorimetric (340 nm) product, proportional to the glutamate present.

### Behavioral Test: Trace Fear Conditioning (TFC)

Sixty-four mice were trained to associate an unconditioned stimulus (foot shock) and a conditioned stimulus (tone) with the environment. For fear conditioning, mice were placed in shock chambers (Coulbourn Instruments) that were scented with 75% EtOH. After 100 s of habituation, the rmice were fear-conditioned with three tone-shock pairings consisting of a 20-s (5 kHz, 80 dB) tone (conditioned stimulus, CS) that coterminated with a 2-s (0.5-mA) foot shock (unconditioned stimulus, US). The intertrial intervals between tone-shock pairings were 100 s. After the final tone-shock pairing, the mice were allowed to remain in the conditioning chambers for 100 s before being returned to their home cages. Mice anticipate the shock by “freezing”, which is defined as the absence of all movement except respiration; this defensive posture reflects learned fear. When placed in the same context on a subsequent occasion, the learned fear is recalled, and the degree of learning and recall can be determined based on the extent of freezing. Contextual memory of the learned fear was assessed 1 day after the treatment, and freezing behavior in the absence of the tone and shock was automatically scored by video tracking software (Xeye Fcs, Beijing Macro Ambition S&T Development Co., Ltd., Beijing, China) over the course of 300 s. The freezing scores for each subject are expressed as percentages of the total testing time.

### qPCR: Quantitative Real-Time Polymerase Chain Reaction

QPCR was conducted according to the methods in our previous study. Total RNA (1 μg) was isolated from the hypothalamus, hippocampus, and MCs using Trizol (Takara, Japan). Total RNA was reverse-transcribed using a PrimeScript RT Reagent Kit (Takara, Japan), and the resulting cDNA was subjected to qPCR in a reaction mixture with SYBR^®^ Green Real-time PCR Master Mix (Takara, Japan) using a real-time PCR system (Applied Biosystems 7500, USA) according to the manufacturer’s instructions. The mouse cytokine-specific primers, NR2B primers, tryptase primers, and GAPDH primers were prepared as follows:

IL-6 mus (forward), 5′TAGTCCTTCCTACCCCAATTTCC3′;

IL-6 mus (reverse), 5′TTGGTCCTTAGCCACTCCTTC3′;

IL-1β mus (forward), 5′GCAACTGTTCCTGAACTCAACT3′;

IL-1β mus (reverse), 5′ATCTTTTGGGGTCCGTCAACT3′;

NR2B mus(forward), 5′GCCATGAACGAGACTGACCC3′;

NR2B mus(reverse), 5′GCTTCCTGGTCCGTGTCATC3′;

GAPDH mus (forward), 5′ATGGCATGGCTTACACCACC3′;

GAPDH mus (reverse), 5′GAGGCCAATTTTGTCTCCACA3′;

When satisfactory primer efficiencies and dissociation curves were obtained, qPCR was run in duplicate in 96-well microtiter plates after 5 min of centrifugation (2,000× rpm). The cycling conditions were as follows: step 1, 95°C (10 min); step 2, 95°C (15 s); step 3, 60°C (60 s); and step 4, 72°C (20 s). Steps 2–4 were repeated 40× and were followed by a dissociation step. qPCR was repeated at least three times per experiment to evaluate whether the results were consistent. The results are shown as the relative expression levels calculated by the 2^−ΔΔCT^ method.

### Western Blot Analysis

The mice were sacrificed after all the *in vivo* experiments were completed. Tissue samples from the hypothalamus and hippocampus were homogenized in ice-cold RIPA buffer combined with 1% phosphatase inhibitor cocktail (KeyGEN) and 1% protease inhibitor cocktail (KeyGEN) and centrifuged at 12,000× rpm for 15 min at 4°C. Proteins were quantified using a BCA kit (Thermo Scientific^TM^) and mixed with an equal volume of loading buffer. Equal amounts of protein samples (approximately 60 μg) were loaded in each well, and one well was loaded with a prestained protein ladder (Invitrogen). Following electrophoresis (120 V for 45 min), the proteins were transferred to PVDF membranes (EMD Millipore). The bands were incubated with primary antibodies against PSD-95 (Proteintech), NR2B (Proteintech), occludin (Abcam), tryptase (Abcam), Tubulin (Proteintech), and GAPDH (Proteintech) at 4°C overnight. Additionally, primary polyclonal rabbit antibodies used for immunoblotting were those against the phosphorylated form of LAT, PI3K, PLCγ1 (Tyr783), Akt (Ser473), p38, JNK, or IKK and those against total LAT, PI3K, or PLCγ1, all of which were from Cell Signaling Technology. After being washed with TBST three times, the membranes were incubated with horseradish peroxidase (HRP)-conjugated secondary antibodies at 1:5,000 for 2 h at room temperature and developed using ECL Prime Western Blotting Detection Reagent (Millipore). Visualization and imaging of the blots were performed with a Bio-Imaging System (Bio-Rad, USA). The experiment was repeated three times.

### Bioinformatics Data Collection

The microarray expression data (generated by the Agilent-014868 Whole Mouse Genome Microarray, GSE64287) from publicly available Gene Expression Omnibus (GEO) database. We selected GSM1568011, GSM1568012, GSM1568013, GSM1568023, GSM1568024 and GSM1568025 for the analysis. We subsequently analyzed the differential expression genes between the control group (WT BMMCs with PBS) and the experimental group (WT BMMCs with 100 ng/ml LPS) by performing GEO2R tool.

### Statistical Analysis

The experimental data are described as the mean ± SD and were analyzed using GraphPad Prism 8 (GraphPad Software, USA). All the data were analyzed with one- or three way analysis of variance (ANOVA) to determine group differences. *P*-values were considered to indicate significance at *p < 0.05, **p < 0.01, and ***p < 0.001.

## Results

## Laparotomy Increases Neuroinflammation and Leads to Cognitive Impairment in Mice in a Manner Dependent on Vagus Partially

**Figure 1:**
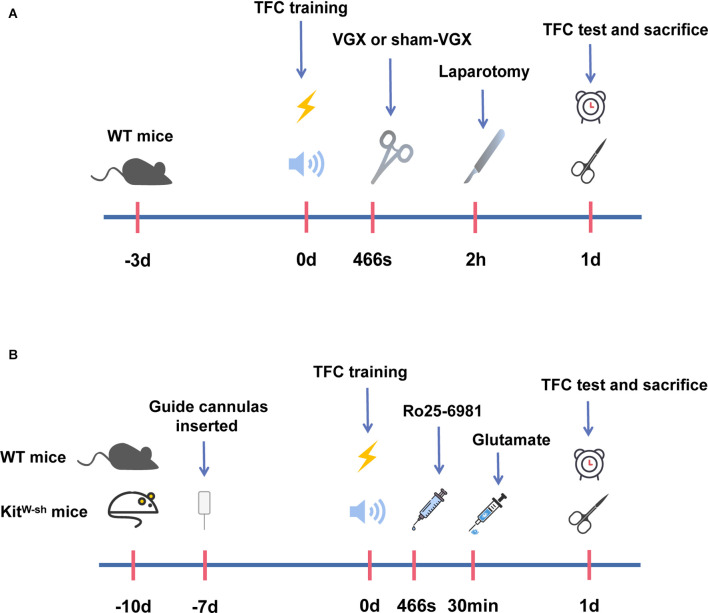
A schematic representation of the operation timeline and administration timeline in mice. **(A)** Mice were acclimatized in the procedure room for the first 3 days. Trace fear conditioning (TFC) training was started on the 4th day. Meanwhile, mice were given vagotomy (VGX) or sham-VGX (expose but do not cut). After 2 h, laparotomies were performed, and behavioral tests were carried out 1 day after laparotomy. One day later, we harvested brain tissue for biochemical and protein expression studies. **(B)** Mice were acclimatized in the procedure room for the first 3 days. On the 4th day, mice were anesthetized and inserted a guide cannula into the right hypothalamus. After 7 days, the mice received TFC training, and then drugs were injected into the right hypothalamus through the implanted guide cannula. Firstly, mice were injected with Ro25-6981, and 30 min later with glutamate. Behavioral tests were performed after 1 day following the glutamate injection on the 11th day of our experimental period. After this, the mice were sacrificed and brain tissues were acquired for the biochemical and protein expression studies 1 day after laparotomy.

**Figure 2:**
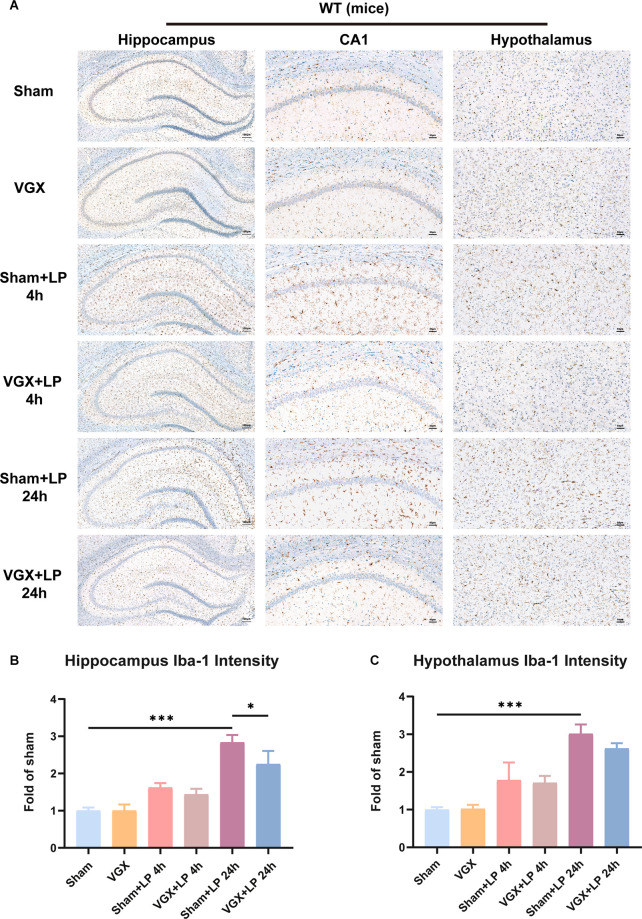
Unilateral cervical vagotomy attenuated the effects of laparotomy (LP) on Iba-1 expression. **(A)** Representative Immunohistochemistry of Iba-1 in the hippocampus and hypothalamus. **(B)** Column graphs representing Iba-1 expression (*n* = 3) in the hippocampus (sham+LP 4 h vs. VGX+LP 4 h, *p* = 0.8504; sham+LP 24 h vs. VGX+LP 24 h, *p* = 0.0322, Three-way ANOVA). **(C)** Column graphs representing Iba-1 expression (*n* = 3) in the hypothalamus (sham+LP 4 h vs. VGX+LP 4 h, *p* = 0.9988 ; sham+LP 24 h vs. VGX+LP 24 h, *p* = 0.4126, Three-way ANOVA). *P* < 0.05 was considered to be statistically significant. *p < 0.05, ***p < 0.001.

**Figure 3:**
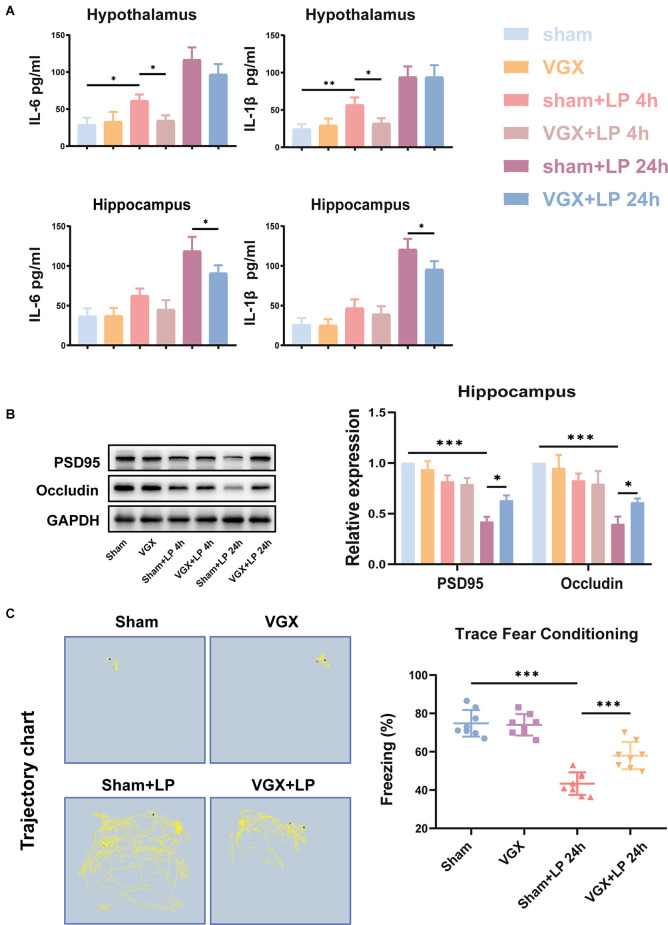
Unilateral cervical vagotomy alleviated LP-induced proinflammatory factors production, blood-brain barrier (BBB) destruction and cognitive decline. **(A)** The levels of the proinflammatory factors IL-6 and IL-Iβ were detected by ELISA. LP significantly increased IL-6 (Hippocampus: sham+LP 4 h vs. VGX+LP 4 h, *p* = 0.3133; sham+LP 24 h vs. VGX+LP 24 h, *p* = 0.0329. Hypothalamus: sham+LP 4 h vs. VGX+LP 4 h, *p* = 0.0494; sham+LP 24 h vs. VGX+LP 24 h, *p* = 0.2225, Three-way ANOVA) and IL-1β (Hippocampus: sham+LP 4 h vs. VGX+LP 4 h, *p* = 0.8867; sham+LP 24 h vs. VGX+LP 24 h, *p* = 0.0243. Hypothalamus: sham+LP 4 h vs. VGX+LP 4 h, *p* = 0.0446; sham+LP 24 h vs. VGX+LP 24 h, *p* = 0.9993, Three-way ANOVA) expression in the hypothalamus or hippocampus 4 h or 24 h later (*n* = 4). **(B)** Protein expressions of PSD-95 (sham+LP 4 h vs. VGX+LP 4 h, *p* = 0.9975; sham+LP 24 h vs. VGX+LP 24 h, *p* = 0.0203, Three-way ANOVA) and Occludin (sham+LP 4 h vs. VGX+LP 4 h, *p* = 0.9930; sham+LP 24 h vs. VGX+LP 24 h, *p* = 0.0179, Three-way ANOVA) in the hippocampuswere examined by Western blotting (*n* = 3). **(C)** Context fear response, as measured by freezing behavior, was determined in the mice (*n* = 8; sham vs. sham+LP 24 h, *p* < 0.001; sham+LP 24 h vs. VGX+LP 24 h, *p* < 0.001, One-way ANOVA). Data are presented as the mean ± SD. *p < 0.05, **p < 0.01, ***p < 0.001.

First, we studied the effects of cervical vagotomy on the activation of microglia, impairment of hippocampal memory, and increases in inflammatory cytokine levels observed in mice followed by laparotomy 2 h later. Cognitive function was subsequently assessed using the TFC test 1 day after laparotomy (Figure 1A). In this study, Iba-1 staining quantification revealed that acute microglial activation in the hypothalamus and hippocampus (Figure 2A) was apparent at 4 h and 24 h after laparotomy. Laparotomy significantly increasedIba-1 intensity in the hypothalamus and hippocampus in the sham + laparotomy (sham+LP) group at 24 h (Figures 2B,C). However, Iba-1 intensity in the hippocampus in the vagotomy + laparotomy (VGX+LP) group was significantly lower than those in the sham+LP group at 24 h after laparotomy (Figure 2B). The morphological analysis of microglia was consistent with the statistical results of Iba-1 staining quantification (Supplementary Figure 1). Morphological analysis of microglia was carried out according to Kimberly’s protocol (Young and Morrison, 2018). ELISA showed that the change trends of the inflammatory factors IL-6 and IL-1β in the hippocampus and hypothalamus were consistent with that of microglia activation (Figure 3A). Occludin, an integral membrane protein that composes tight junction strands, contributes to BBB integrity. We next examined the expression levels of PSD95 and occludin using western blotting and found that they were significantly lower at 24 h in the sham+LP group than in the VGX+LP group (Figure 3B). Additionally, there was no difference in the expression of tight junction proteins between the two groups at 4 h, and the expression levels were similar to the sham group. This result showed that the BBB had not yet been damaged 4 h after laparotomy, so the early neuroinflammation after laparotomy may be largely caused by other pathways (such as neural pathways).

As shown in Figure 3C, the mice who underwent laparotomy exhibited a significant reduction in cognitive function, as indicated by a reduction in freezing behavior compared to those subjected to both vagotomy and laparotomy. Thus, laparotomy-induced inflammation in the periphery is associated with increased neuroinflammation. Interestingly, vagotomy can partially block this process.

## Laparotomy Can Activate Hypothalamic Glutamatergic Neurons and Increase the Hypothalamic Glutamate Levels Through the Vagus Nerve

**Figure 4:**
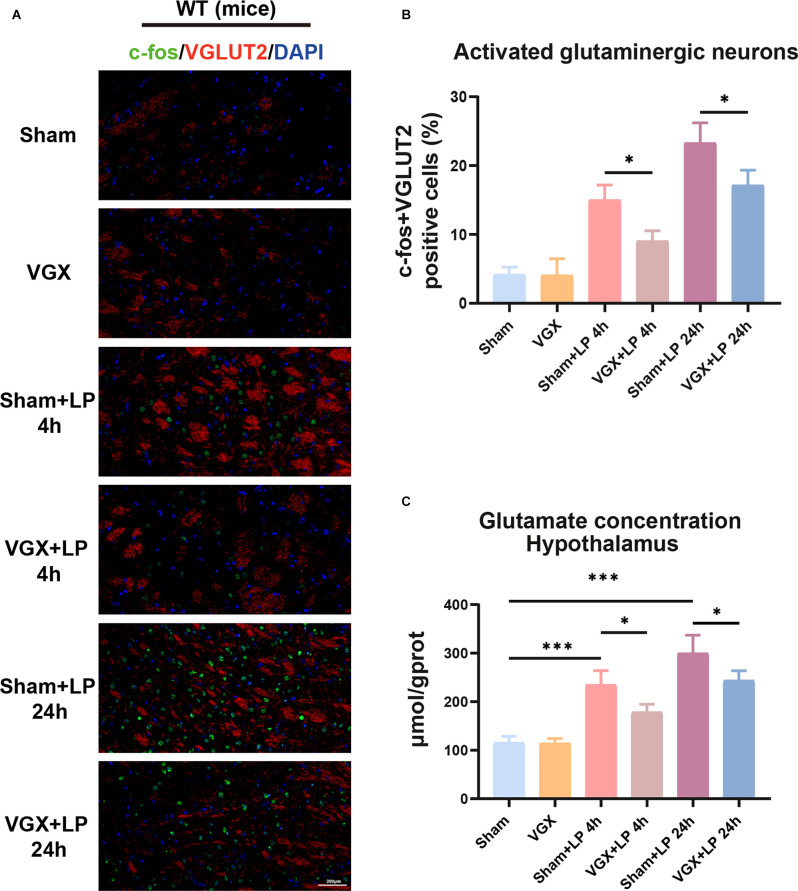
Laparotomy induced activation of glutaminergic neurons and elevation of glutamate levels. **(A)** Immunofluorescence staining showed that the c-fos positive cells were co-localized with some VGLUT2 positive neurons. **(B)** Quantification of activated glutaminergic neurons (*n* = 3; sham+LP 4 h vs. VGX+LP 4 h, *p* = 0.0420; sham+LP 24 h vs. VGX+LP 24 h, *p* = 0.0356, Three-way ANOVA). **(C)** Glutamate concentration of hypothalamus was determined by Glutamate Assay Kit (*n* = 4; sham+LP 4 h vs. VGX+LP 4 h, *p* = 0.0253; sham+LP 24 h vs. VGX+LP 24 h, *p* = 0.0276, Three-way ANOVA). *p < 0.05, ***p < 0.001.

The *in vivo* experiments revealed that laparotomy promoted activation of glutamate neurons and elevation of glutamate levels in the hypothalamus, which could be suppressed by the vagotomy. In Figure 4, neurons co-stained with c-fos and VGLUT2 were used to represent activated glutaminergic neurons. We observed that mice with prior vagotomy exhibited reduced glutamatergic neurons activation (Figures 4A,B) and decreased glutamate concentrations at 4 h after laparotomy (Figure 4C). Vagotomy itself did not increase glutamate concentrations. Similarly, at 24 h, there was a statistically significant difference in glutamatergic neurons activation and glutamate concentration between the sham+LP group and the VGX+LP group.

## Laparotomy Can Activate BPMCs and MMCs Through the Vagus Nerve, Vagotomy Can Partially Inhibit This Activation at an Early Stage After Laparotomy

**Figure 5:**
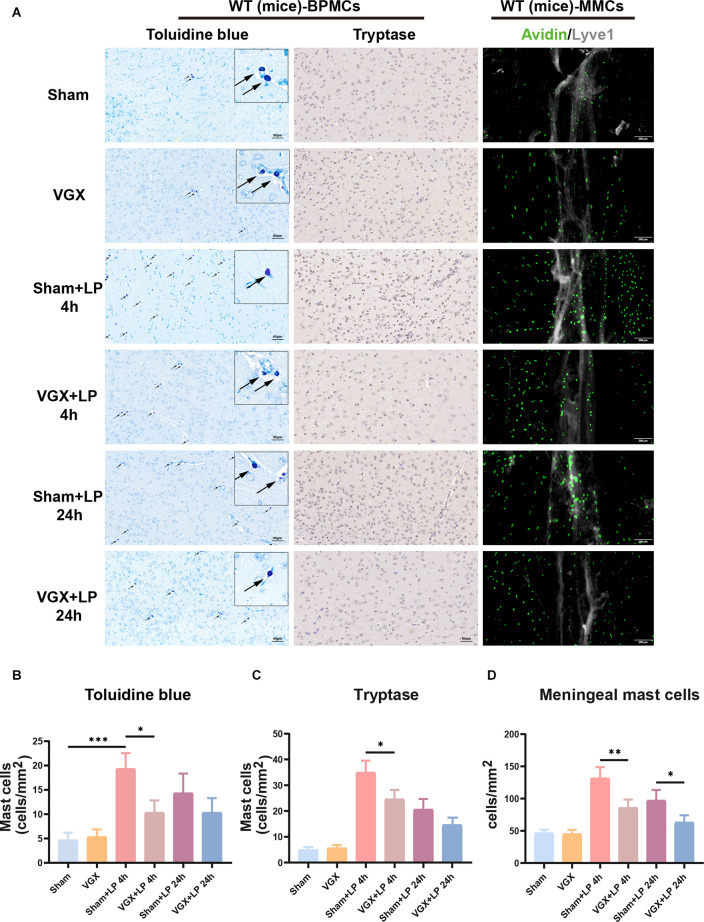
Unilateral cervical vagotomy repressed LP-induced brain mast cell activation (include BPMCs and MMCs). **(A)** BPMCs were stained with toluidine blue and tryptase, while MMCs were stained with avidin. **(B,C)** Quantification of activated BPMCs (*n* = 3; **B**: sham+LP 4 h vs. VGX+LP 4 h, *p* = 0.0200; sham+LP 24 h vs. VGX+LP 24 h, *p* = 0.5600. C: sham+LP 4 h vs. VGX+LP 4 h, *p* = 0.0154; sham+LP 24 h vs. VGX+LP 24 h, *p* = 0.2448, Three-way ANOVA). **(D)** Quantification of activated MMCs (*n* = 3; sham+LP 4 h vs. VGX+LP 4 h, *p* = 0.0061; sham+LP 24 h vs. VGX+LP 24 h, *p* = 0.0439, Three-way ANOVA). BPMCs, brain parenchymal mast cells; MMCs, meningeal mast cells. *p < 0.05, **p < 0.01, ***p < 0.001.

We next examined the role of the vagus nerve in mediating peripheral inflammation-triggered activation of BPMCs and MMCs in mice undergoing laparotomy. Toluidine blue staining and tryptase immumohistochemical staining showed that compared with the sham procedure, laparotomy significantly increased the number of BPMCs in the hypothalamus at 4 h following laparotomy (Figures 5A–C). This effect was markedly diminished in mice with cervical unilateral vagotomy, indicating a role of the afferent vagus nerve. In agreement with the above results, MMCs were abundantly observed in the sham+LP group, whereas only limited numbers were seen in the VGX+LP group (Figures 5A,D). In our research, MMCs were marked by avidin staining. Collectively, these data indicate that BMCs (include BPMCs and MMCs) may play a role in mediating the accelerative effect of peripheral inflammation on neuroinflammation in a vagus nerve-dependent manner.

## Bioinformatics Analysis Reveals That the NR2B Receptor may Play an Important Role in Mediating BMMCs-Induced Inflammation

### Functional Enrichment Analysis

**Figure 6:**
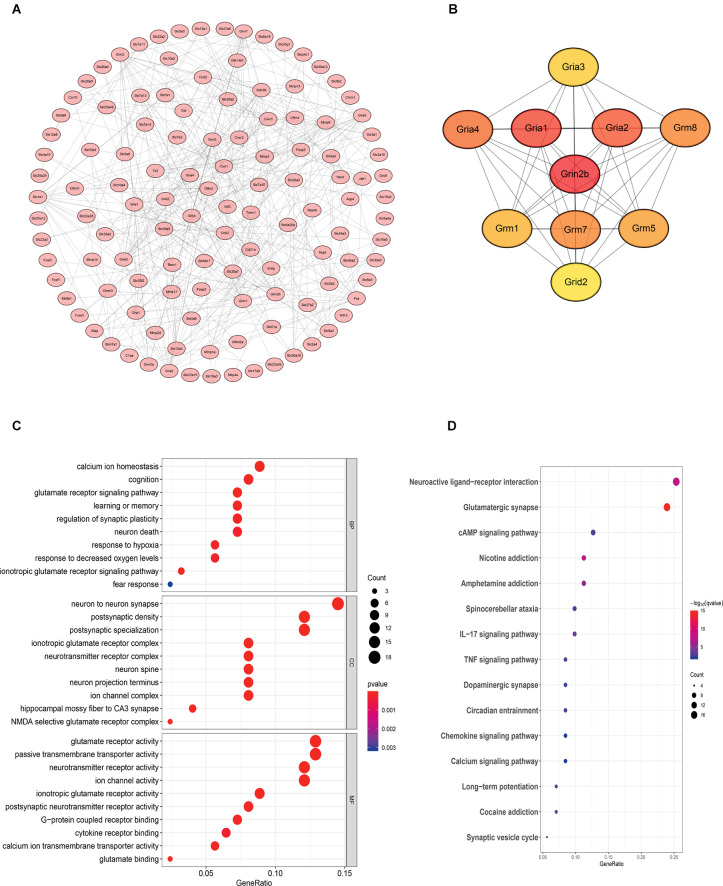
Bioinformatics analysis reveals that the NR2B receptor and glutamate receptor signaling pathway may play an important role in mediating neuroinflammation. The samples were all derived from bone marrow derived mast cells (BMMCs). **(A)** A PPI network was constructed from STRING using the 864 common DEmRNAs; **(B)** PPI network of 10 hub genes extracted from **(A)**. Enrichment of top 10 GO terms **(C)** and KEGG pathways **(D)** of differentially expressed mRNAs. The node color changes gradually from blue to red in ascending order according to the adjusted *p*-values. The size of the node represents the number of counts.

Gene Ontology (GO) functional enrichment and Kyoto Encyclopedia of Genes and Genomes (KEGG) pathway analyses were performed to explore the functions of genes of interest. The results revealed that a total of 178 GO terms and 26 KEGG pathways were significantly enriched. The top 10 significant GO terms are shown in Figure 6; the genes of interest were mainly enriched for the “calcium ion homeostasis”, “cognition” and “glutamate receptor signaling pathway” terms in the biological process (BP) category; the “neuron to neuron synapse” and “postsynaptic density” terms in the cellular component (CC) category; and the “glutamate receptor activity” and “passive transmembrane transporter activity” terms in the molecular function (MF) category. Moreover, the top 10 pathway terms related to the genes of interest in KEGG analysis included the “neuroactive ligand-receptor interaction”, “glutamatergic synapse” and “cAMP signaling pathway” terms.

### Protein-Protein Interaction (PPI) Network Construction and Hub Gene Identification

To further investigate the interactions among the genes of interest, we established a PPI network (Figure 6). The PPI network contained 218 nodes and 356 edges. Moreover, the maximal clique centrality (MCC) algorithm in Cytoscape was conducted to screen hub genes in the PPI network. Based on the MCC scores, the top ten highest-scoring genes, including Grin2b (NR2B), Gria1, Gria2, Gria4, Grm8, Grm7, Grm5, Grm1, Gria3, and Grid2, were identified as hub genes. Among them, NR2B is the core gene.

### Intrahypothalamic Injection of Glutamate Increases Neuroinflammation and Cognitive Dysfunction, Which Is Attenuated by NR2B Receptor Antagonist Treatment or MCs Knockdown

**Figure 7:**
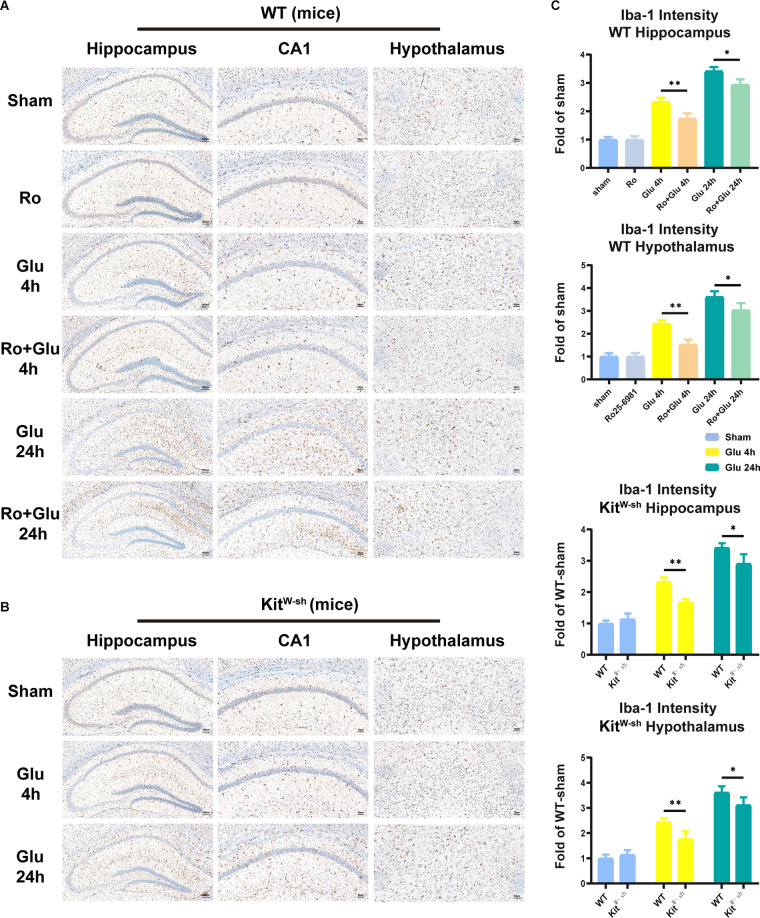
Mast cell (MC) deficiency or NR2B receptor antagonist repressed glutamate-induced microglia activation in the hippocampus and hypothalamus. **(A,B)** Immunohistochemical analysis was used to detect Iba1 expression. The effect of glutamate on microglia activation was decreased in mast cell-deficient Kit^W-sh^ mice. The activated microglia cells had larger cell bodies, poorly ramified short and thick processes. **(C)** Quantification of Iba1 intensity (*n* = 3; Hippocampus: WT-Glu 4 h vs. WT-Ro+Glu 4 h, *p* = 0.0043; WT-Glu 24 h vs. WT-Ro+Glu 24 h, *p* = 0.0194; WT-Glu 4 h vs. Kit^W-sh^-Glu 4 h, *p* = 0.0040; WT-Glu 24 h vs. Kit^W-sh^-Glu 24 h, *p* = 0.0100. Hypothalamus: WT-Glu 4 h vs. WT-Ro+Glu 4 h, *p* = 0.0019; WT-Glu 24 h vs. WT-Ro+Glu 24 h, *p* = 0.0457; WT-Glu 4 h vs. Kit^W-sh^-Glu 4 h, *p* = 0.0060; WT-Glu 24 h vs. Kit^W-sh^-Glu 24 h, *p* = 0.0200, Three-way ANOVA). *p < 0.05, **p < 0.01.

To provide deeper insight into the possibility that BMCs and glutamate can communicate in the inflamed brain, intrahypothalamic stereotactic injection of glutamate was performed in WT and Kit^W-sh^ mice, respectively. The mice were administered an injection of saline (1 μl) or Ro25-6981 (1 μM, 1 μl) in the right hypothalamus, followed by glutamate (500 μM, 1 μl) or saline (1 μl) injection 30 min later. Cognitive function was subsequently assessed using the TFC test 1 day after administration (Figure 1B). Iba-1 staining quantification and morphological analysis showed that microglial activation was significantly increased in the hypothalamus and hippocampus at both 4 h and 24 h following glutamate administration (i.h.t., 500 μM glutamate/1 μl), while microglial activation was significantly lower in the WT-Ro+Glu group (i.h.t., 1 μM Ro/1 μl+500 μM glutamate/1 μl) and Kit^W-sh^-Glu group (i.h.t., 500 μM glutamate/1 μl) than in the WT-Glu group (i.h.t., 500 μM glutamate/1 μl; Figure 7, Supplementary Figure 2).

**Figure 8:**
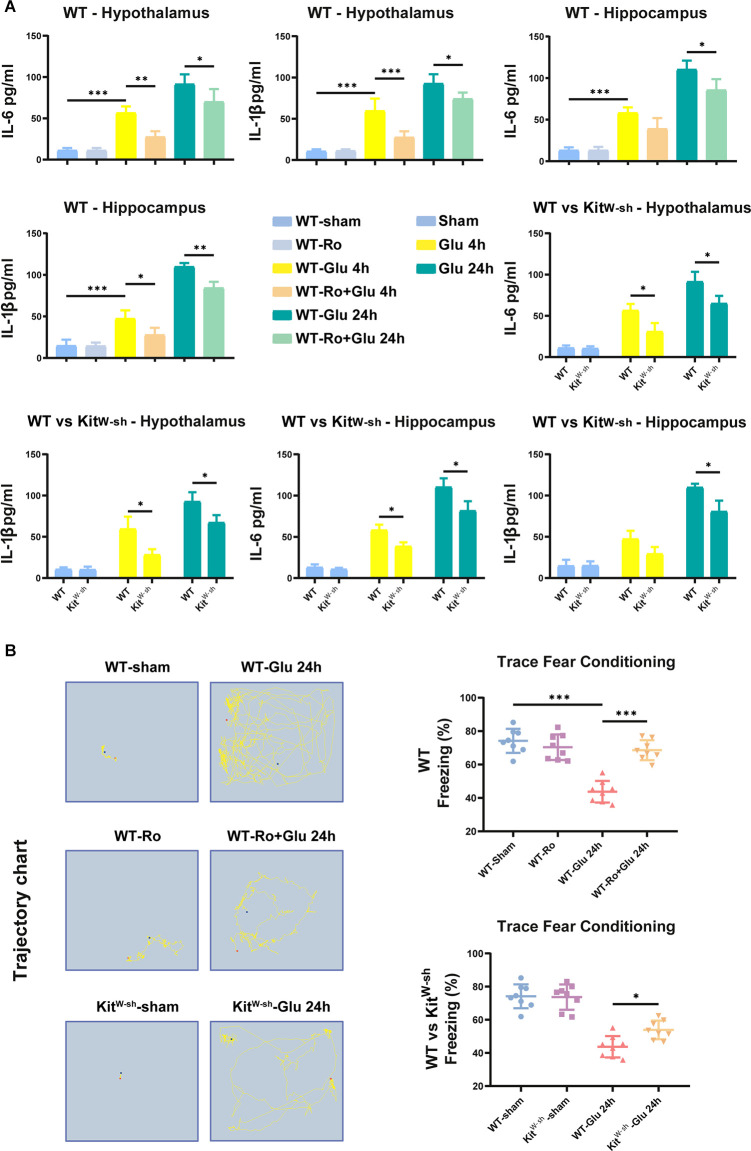
MC deficiency or NR2B receptor antagonist-Ro reduced glutamate-induced proinflammatory factors production, BBB destruction, and cognitive decline in mice. **(A)** ELISA results showed glutamate significantly increased IL-6 (Hippocampus: WT-Glu 4 h vs. WT-Ro+Glu 4 h, *p* = 0.0787; WT-Glu 24 h vs. WT-Ro+Glu 24 h, *p* = 0.0241; Hypothalamus: WT-Glu 4 h vs. WT-Ro+Glu 4 h, *p* = 0.0032; WT-Glu 24 h vs. WT-Ro+Glu 24 h, *p* = 0.0346, Three-way ANOVA) and IL-1β (Hippocampus: WT-Glu 4 h vs. WT-Ro+Glu 4 h, *p* = 0.0227; WT-Glu 24 h vs. WT-Ro+Glu 24 h, *p* = 0.0087; Hypothalamus: WT-Glu 4 h vs. WT-Ro+Glu 4 h, *p* = 0.0009; WT-Glu 24 h vs. WT-Ro+Glu 24 h, *p* = 0.0378, Three-way ANOVA) expression in the hypothalamus or hippocampus 4 h or 24 h later in WT mice (*n* = 4). While in Kit^W-sh^ mice or Ro pre-treating mice, this increase of IL-6 (Hippocampus: WT-Glu 4 h vs. Kit^W-sh^-Glu 4 h, *p* = 0.0135; WT-Glu 24 h vs. Kit^W-sh^-Glu 24 h, *p* = 0.0324. Hypothalamus: WT-Glu 4 h vs. Kit^W-sh^-Glu 4 h, *p* = 0.0289; WT-Glu 24 h vs. Kit^W-sh^-Glu 24 h, *p* = 0.0461, Three-way ANOVA) and IL-1β (Hippocampus: WT-Glu 4 h vs. Kit^W-sh^-Glu 4 h, *p* = 0.0911; WT-Glu 24 h vs. Kit^W-sh^-Glu 24 h, *p* = 0.0468. Hypothalamus: WT-Glu 4 h vs. Kit^W-sh^-Glu 4 h, *p* = 0.0313; WT-Glu 24 h vs. Kit^W-sh^-Glu 24 h, *p* = 0.0390, Three-way ANOVA) was not as obvious as the above. **(B)** Effects on the occurrence of cognitive impairment in TFC task (*n* = 8; WT-Glu 24 h vs. WT-Ro+Glu 24 h, *p* < 0.001; WT-Glu 24 h vs. Kit^W-sh^-Glu 24 h, *p* = 0.0300, One-way ANOVA). Data are presented as the mean ± SD. *p < 0.05, **p < 0.01, ***p < 0.001.

The changes in hippocampal and hypothalamic levels of IL-6 and IL-1β were consistent with the activation of microglia (Figure 8A). Similarly, prior Ro treatment and MCs knockdown significantly attenuated the glutamate-induced increases in freezing time in the TFC test (Figure 8B). These results suggest that BMCs play an important role in neuroinflammation induced by glutamate.

### Intrahypothalamic Injection of Glutamate Activates BPMCs and MMCs in WT Mice, and RO Partially Reverses This Effect

**Figure 9:**
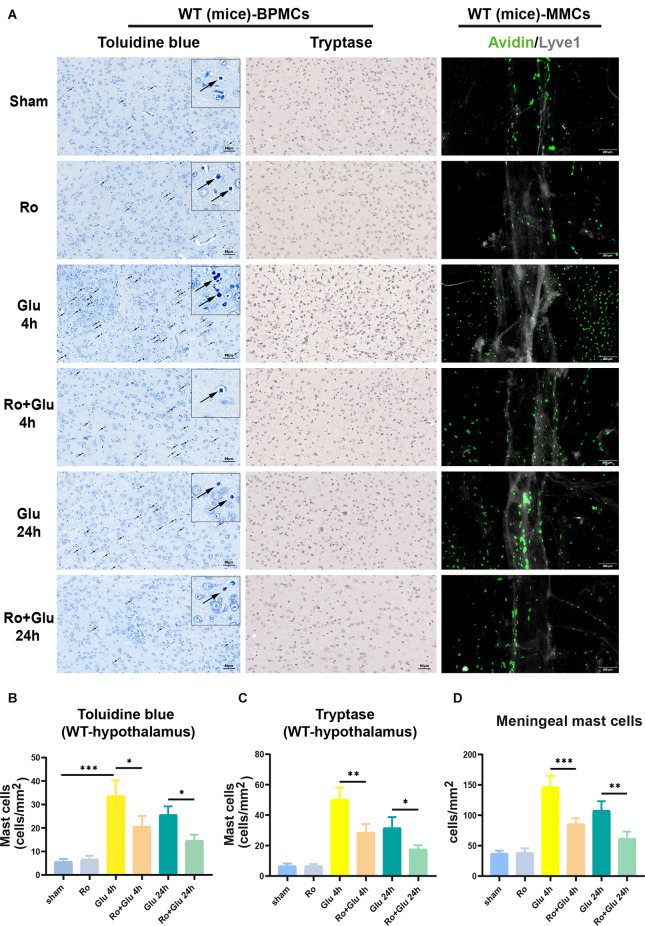
NR2B receptor antagonist-Ro inhibited glutamate-induced BMCs activation in WT mice. **(A,B)** The toluidine blue positive BPMCs (WT-Glu 4 h vs. WT-Ro+Glu 4 h, *p* = 0.0100; WT-Glu 24 h vs. WT-Ro+Glu 24 h, *p* = 0.0400, Three-way ANOVA) and **(A,C)** the tryptase-positive BPMCs (WT-Glu 4 h vs. WT-Ro+Glu 4 h, *p* = 0.0020; WT-Glu 24 h vs. WT-Ro+Glu 24 h, *p* = 0.0444, Three-way ANOVA) in the hypothalamus were significantly increased in glutamate treated mice for 4 h. While in Ro pre-treating mice, the positive rates were significantly decreased (*n* = 3). **(D)** Immunofluorescence analysis was used to detect avidin, markers of mast cells, expression in mice meninges (*n* = 3; WT-Glu 4 h vs. WT-Ro+Glu 4 h, *p* = 0.0005; WT-Glu 24 h vs. WT-Ro+Glu 24 h, *p* = 0.0049, Three-way ANOVA). *p < 0.05, **p < 0.01, ***p < 0.001.

Activation of BPMCs and MMCs was greater in the Glu group than in the sham group, but prior treatment with Ro significantly suppressed the glutamate-induced increase (Figure 9A). The results of toluidine blue staining and tryptase immumohistochemical staining showed that compared to the WT-sham group, the WT-Glu group exhibited significant activation of BPMCs in the hypothalamus at 4 h following laparotomy, but the activation diminished at 24 h (Figures 9A–C). Additionally, immunofluorescence staining suggested that, in comparison with the WT-sham group, the WT-Glu group exhibited more avidin-stained MMCs, while the Ro+Glu group exhibited fewer avidin-stained MMCs (Figures 9A,D). We further examined the tryptase and NR2B levels by western blot analysis. The data showed that both NR2B and tryptase levels were significantly increased at 4 h after glutamate treatment (Supplementary Figure 3). Moreover, the protein levels of PSD95 and Occludin were significantly decreased at 24 h in the WT-Glu group compared with the WT-Ro+Glu group (Supplementary Figure 3). These results indicate that Ro treatment *via* stereotactic injection attenuates glutamate-induced neuroinflammation in mice. Based on the above results, we speculate that glutamate induces neuroinflammation through the functional glutamate-glutamate receptor axis, in which NR2B plays an important role; thus, Ro can alleviate this process.

### Glutamate Induces BMMCs Activation and Inflammatory Cytokine Release Through LAT- PLCγ1 and LAT-PI3K/AKT/MAPK Pathways *In vitro*, but These Effects Are Inhibited by NR2B Receptor Antagonist Treatment

**Figure 10:**
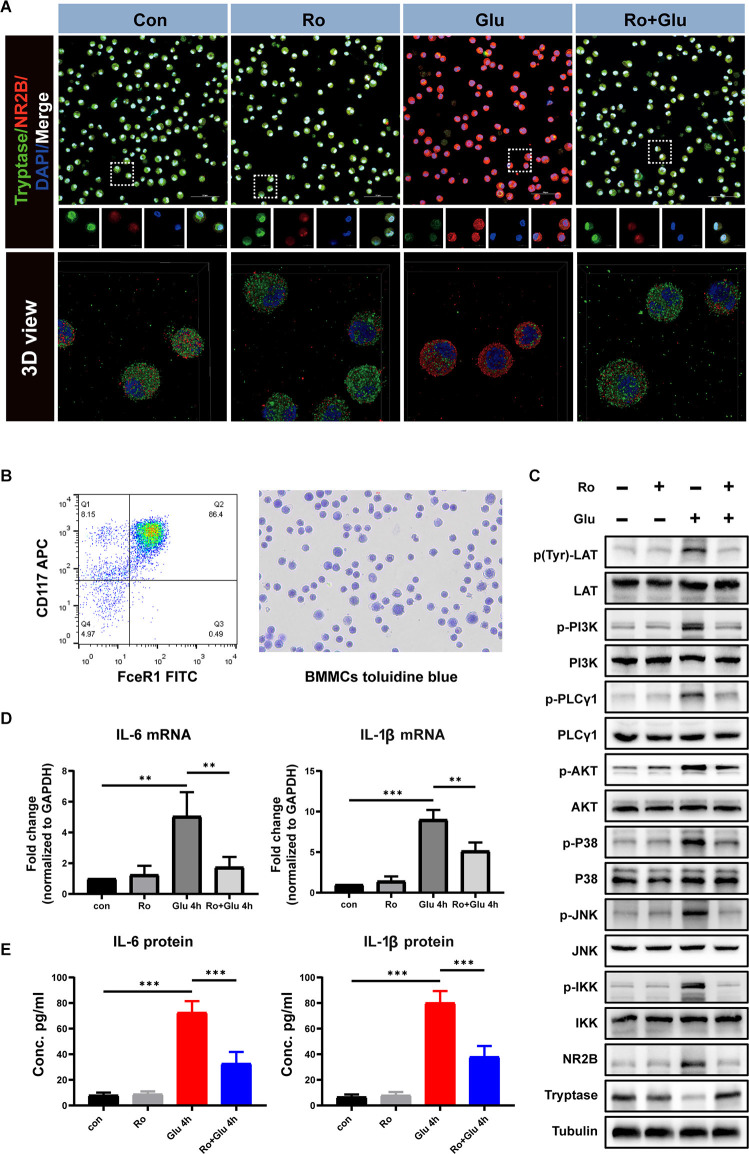
An agonist of NR2B inhibits glutamate-induced BMMCs activation and associated inflammatory response. **(A)** Primary BMMCs were double-stained with tryptase antibody and NR2B antibody (*n* = 3). The 3D reconstruction of the confocal images showed that Ro can significantly reduce glutamate-induced BMMCs degranulation and increase NR2B expression. Blue staining represents DAPI. Green staining represents tryptase. Red staining represents NR2B. **(B)** Flow cytometry and toluidine blue were used to identify the purity of BMMCs. **(C)** Phospho-LAT triggers PLCγ and PI3K phosphorylation followed by a chain of downstream signaling protein activation (phosphorylation). The processes lead to degranulation and mediator release of BMMCs. **(D)** Q-PCR analysis of the relative expression of IL-6 and IL-1β mRNA (*n* = 3) (IL-6: con vs. Glu 4 h, *p* = 0.002; Glu 4 h vs. Ro+Glu 4 h, *p* = 0.009. IL-1β: con vs. Glu 4 h, *p* < 0.001; Glu 4 h vs. Ro+Glu 4 h, *p* = 0.003, One-way ANOVA). Each value was expressed relative to that in the control group, which was set to 1. **(E)** Concentration of IL-6 and IL-1β measured by ELISA (*n* = 3; IL-6: con vs. Glu 4 h, *p* < 0.001; Glu 4 h vs. Ro+Glu 4 h, p *=* < 0.001. IL-1β: con vs. Glu 4 h, *p* < 0.001; Glu 4 h vs. Ro+Glu 4 h, *p* < 0.001, One-way ANOVA). **p < 0.01, ***p < 0.001.

We next assessed the effects of glutamate (100 μM) and Ro (1 μM) on BMMCs *in vitro*. The purity of BMMCs was determined by flow cytometry and toluidine blue staining (Figure 10B). First, immunofluorescence staining showed that NR2B expression was significantly increased at 4 h *in vitro* after glutamate treatment, while Ro treatment significantly prevented this increase (Figure 10A).

Phosphorylation of phosphatidylinositol 3-kinase (PI3K)/Akt/MAPK is the predominant signaling transduction pathway responsible for the production of proinflammatory mediators in mast cell activation (Chang et al., 2020). Phosphorylation of linker for activation of T cells (LAT) and PLCγ1 are the classic pathways that cause mast cell degranulation. We investigated whether Ro could affect Glu-induced phosphorylation of LAT, PI3K, and PLCγ1 phosphorylation. As shown in Figure 10C, treatment with Glu for 4 h induced LAT, PI3K, and PLCγ1 phosphorylation, which could be partially inhibited by Ro (200 μg) administration 30 min in advance. These results indicate that stabilization NR2B receptors on BMMCs suppress Glu-induced LAT-PLCγ1 and LAT-PI3K/AKT/MAPK signaling pathway activation.

Similarly, Glutamate significantly increased the expression of IL-6 and IL-1β at the genetic and protein levels, while Ro effectively prevented these increases (Figures 10D,E). These results suggest that Ro preserves glutamate-induced increases in expression of the ionotropic glutamate receptor NR2B and inflammation in BMMCs.

## Discussion

**Figure 11:**
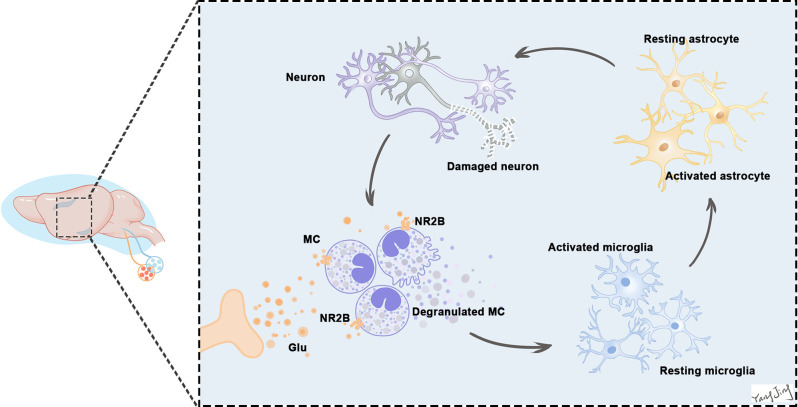
Excessive release of glutamate activates NR2B in BMCs in the earliest stage. BMCs activation leads to the release of many neuropeptides and inflammatory mediators, including histamine, tryptase, and prostaglandins, which can act directly on neurons to cause neuronal damage or through glia (mostly microglia and astrocytes) to aggravate the inflammatory response and eventually damage neurons. Neurons and glias in turn release various neuropeptides and neuroinflammatory and analgesic mediators that can activate BMCs in a vicious cycle. This continuous process leads to increased neuroinflammation. Because of the vicious positive feedback mechanism of BMCs and glial cells activation with inflammatory mediator release, even a small number of BMCs can induce significant neuroinflammation in the brain.

Our key findings were as follows:

(1) The levels of tryptase, glutamate concentration, and BMCs numbers were significantly increased approximately 4 h after laparotomy. Fortunately, vagotomy partially blocked the increases and reduced neuroinflammation caused by peripheral inflammation during the acute phase.
(2) By 4 h after treatment, administration of glutamate to WT mice had significantly increased MC degranulation, microglial activation, and the expression of tryptase, IL-6, and IL-1β. These effects were reversed by the selective NR2B inhibitor-Ro. Utilizing the same method, we found that the behavioral responses of WT mice 24 h after administration of glutamate were significantly worse than the Kit^W-sh^ mice, indicating that BMCs (include BPMCs and MMCs) play an important role in glutamate-induced neuroinflammation. Combined, our data indicate that early activation of BMCs exacerbated neurological impairments and hippocampal neuronal damage after laparotomy.
(3) Glutamate significantly increased tryptase, NR2B, IL-6, and IL-1β expression in BMMCs *in vitro*, consistent with the *in vivo* results. The proinflammatory effects were offset by the NR2B inhibitor Ro. These results suggest that the neuroinflammatory effects of glutamate were partly mediated by activation of glutamate-NR2B signaling. We demonstrated the process with a pattern in which glutamate activated NR2B receptors on BMCs, thereby amplifying the inflammatory cascade (Figure 11).
(4) Furthermore, to the best of our knowledge, we discovered for the first time that the underlying mechanisms of NR2B were associated with LAT- PLCγ1 and LAT-PI3K/AKT/MAPK intracellular signaling in BMMCs.

Here, we show that the vagus nerve mediates peripheral-to-central (P-to-C) inflammation transmission primarily during the acute phase, and that blocking vagus nerve signaling *via* vagotomy reduced inflammatory cytokine levels, MCs degranulation, and microglial activation *in vivo*. Moreover, in our study, NR2B positively regulated inflammation and was upregulated in activated BMCs both *in vivo* and *in vitro*. The NR2B antagonist (Ro) partially reversed the activation of glutamate-treated BMCs, decreased the expression of microglial activation markers, and ultimately improved cognitive function in mice with neuroinflammation.

Recent studies have demonstrated that afferent vagus nerve signaling reaches the forebrain (hippocampus and cortex) and have identified basal forebrain cholinergic nuclei, including the medial septum, as major relay components (Broncel et al., 2018; Suarez et al., 2018). A gut-to-brain neural circuit indicates that the vagus is an essential component of the neuronal reward pathway, linking sensory neurons in the upper gut to striatal dopamine release (Han et al., 2018). In addition, Kevin J. Tracey and Valentin A. Pavlov recently found that signaling through the vagus nerve mediates brain cholinergic modulation of peripheral inflammation (Suarez et al., 2018). Such regulation possibly involves other brain regions through a multisynaptic pathway, with hypothalamic nuclei receiving peripheral vagus nerve projections. However, such brain networks remain to be elucidated. Our study also demonstrated the importance of the vagus nerve in P-to-C communication. The vagus nerve transmits peripheral immune inflammation-related information to the CNS directly or through the mNTS. Subsequently, signals from mNTS are further relayed to the hypothalamus, which is projected upwards to the hippocampus and cerebral cortex. Such as circuit may be helpful for the cortex to issue commands and regulate peripheral reactions.

In the process of P-to-C inflammation, both the nervous and immune systems play critical roles. As archetypal neuroimmune cells, BPMCs are essential intermediaries between the immune and nervous systems and function in both systems (Xu et al., 2020). BPMCs and neurons are closely associated both anatomically and functionally throughout the body, including in the CNS (Forsythe, 2019). BPMCs are the first responders to brain injuries since BPMCs can release prestored mediators. They can act indirectly *via* interactions with glial cells and neurons by releasing signaling molecules such as IL-6, IL-1β, and nitric oxide (NO), and also directly *via* the release of mediators (e.g., TNF-α, histamine, chymase; Zhang et al., 2016). The number and distribution of BPMCs in the brain can change during infection, trauma, or stress (Bugajski et al., 1994; Maslinska et al., 2005; Silver and Curley, 2013). At present, BPMCs are implicated in many neurological pathologies, such as brain injuries, stress, neuroinflammation, traumatic brain injury (TBI), stroke, multiple sclerosis (MS), experimental autoimmune encephalomyelitis (EAE), Alzheimer’s disease (AD), and neuropathic pain (Kempuraj et al., 2017).

In addition to being the initial responders in various neurological pathologies, BPMCs also disrupt BBB, enabling entry of toxins and immune cells and exacerbation of the inflammatory microenvironment. In our study, we found that activation of BPMCs after laparotomy occurs before activation of other immune cells. Therefore, as the first line of defense in brain immunity, BPMCs are important in mediating P-to-C inflammatory. However, at present, mechanisms underlying the relationship between BPMCs and neuroinflammation are elusive. Recently, the effects of neurotransmitters and neuropeptides on BPMCs have attracted our attention. Because the neural pathway is always faster than other physiological response pathways, we believe that some neurotransmitters may enable communication between the fast neuroimmune pathway and BPMCs.

Glutamate is a very important excitatory neurotransmitter in the CNS. Under pathological conditions, it can produce excitotoxicity, which is now recognized as an important pathological mechanism in many neurological disorders. Trauma, poisoning, ischemia, hypoxia, and other factors can cause the excessive release and abnormal accumulation of glutamate in the central nervous system, resulting in acute cell swelling. Ca^2+^ overload and release of large amounts of NO and free radicals lead to neuronal degeneration and death. Shortly following acute injury, glutamate levels increase by 50-fold, sufficient to lead to cell death (Culley et al., 2004). In addition, cells can be damaged by physiological concentrations of glutamate under pathological conditions. Recently, Li et al. (2015) found that glutamate imbalance is involved in POCD in aged mice. Considering the important role of glutamate in other Neurological disorders, the involvement of glutamate abnormalities in the occurrence of POCD cannot be ignored. A recent article on glutamate activation of mast cells and production of inflammation has attracted our attention (Alim et al., 2017). Because both glutamate and BMCs are fast responders to insults, we speculated that early activation of BMCs may cause abnormality of glutamate, which amplifies the neuroinflammation and leads to POCD. Such a speculation was confirmed in our work. Still, how the released glutamate from the terminal end of the sensory central axon of the vagus activates glutamatergic neurons is still unknown. Further research is needed to explore the specific mechanism in the process in the future.

Ionotropic NMDA receptors of glutamate are involved in a variety of physiological and pathological activities of the CNS. Some studies have shown that the NR2B subunit is highly expressed in the hypothalamic paraventricular nucleus (PVN) and the supraoptic nucleus neurons, and participates in the regulation of hypothalamic-pituitary-adrenal (HPA) axis excitability (Chen et al., 2008; Sheng et al., 2008). Furthermore, Radulovic et al. (2019) have reported that the expression of NR2B in the thalamus is higher than that of other NMDA receptors. When the expression of NR2B in the brain is abnormally increased, the NMDA receptor is overactivated, and excitatory neurotoxicity is significantly increased (Vizi et al., 2013; Moskal et al., 2014). The NR2B subunit directly regulates hippocampal synaptic long-term potentiation and inhibition, which is critical for cognitive functions such as learning and memory (Autry et al., 2011; Volianskis et al., 2013; Wyllie et al., 2013). In addition, some studies have found that the protein and nucleic acid expression of NR2B increases after inflammatory brain injury, which is critical in mediating excitatory neurotoxicity. The resulting neurotoxicity induces long-term synaptic inhibition in the hippocampus, impairing cognitive function (Kamat et al., 2014). In summary, we found that NR2B can directly regulate synaptic plasticity in the hippocampus and affect synaptic long-term potentiation and inhibition, both of which are indispensable for the maintenance and improvement of cognitive function. However, it can also mediate excitatory neurotoxic injury under pathological conditions, causing neuronal necrosis and apoptosis that lead to cognitive dysfunction and memory impairment (Monaco et al., 2015).

In this study, we found that glutamate-induced neuroinflammation was stronger in WT mice than in Kit^W-sh^ mice, and that application of Ro, an NR2B inhibitor, reversed this effect in WT mice. Therefore, it is possible that NR2B is upregulated, as a positive feedback mechanism to enhance neuroinflammation in response to the overwhelming degranulation of BMCs. NR2B receptors play essential roles in cognition, memory, neuroprotection, and inflammation, suggesting that it is a potential therapeutic target in the aforementioned pathology. One drawback in our study is that we don’t pay much attention to the role of other glutamate receptors. We look forward to more studies on the specific role and mechanism of other receptors in the occurrence and development of neuroinflammation and POCD.

Together with our previous findings, our observations suggest that the vagus nerve is one of the main pathways by which glutamate activates BMCs in the acute phase. Moreover, the BMC-neurotransmitter interaction is the key point at which the brain senses peripheral inflammatory signals at an early stage. In the future, more *in vitro* models are needed to elucidate the mechanism of crosstalk between BMCs and various neural signals.

## Conclusion

This study investigated the mechanism of peripheral inflammation in activating central neuroinflammation. Importantly, the vagus nerve appears to be a fast route by which peripheral signals transmit to the brain. Additionally, peripheral inflammation led to increased levels of glutamate and glutamate receptor subunit NR2B in the brain, which activated BMCs and amplified neuroinflammation. Thus, the vagus nerve served as a tie between the PNS and the CNS. We believe that agents targeting the neurotransmitter receptors on MCs will be of significant therapeutic value.

## Data Availability Statement

The original contributions presented in the study are included in the article/Supplementary Materials, further inquiries can be directed to the corresponding author/s.

## Ethics Statement

The animal study was reviewed and approved by The Institutional Animal Care and Use Committee (IACUC) of Nanjing Medical University (No. 1911004).

## Author Contributions

JY: conception, design, implementation of the experiment, analysis of data, interpretation of data, drafting, revising content of manuscript, and final approval. H-QD: designed the studies, revising content of manuscript, and final approval. Y-HL: implementation of the experiment, interpretation of data, and final approval. M-HJ and XZ: revising content of manuscript and final approval. H-YD, LL, JZ, Z-CS, and H-HS: implementation of the experiment and final approval. Y-NQ and Q-GL: revising content of manuscript and final approval. HY: conception, revising content of manuscript and final approval. N-NL: conception, design, implementation of the experiment, revising content of manuscript, and final approval. All authors contributed to the article and approved the submitted version.

## Conflict of Interest

The authors declare that the research was conducted in the absence of any commercial or financial relationships that could be construed as a potential conflict of interest.

## Publisher’s Note

All claims expressed in this article are solely those of the authors and do not necessarily represent those of their affiliated organizations, or those of the publisher, the editors and the reviewers. Any product that may be evaluated in this article, or claim that may be made by its manufacturer, is not guaranteed or endorsed by the publisher.

## Supplementary Materials

Supplementary Figure 1 **(A,B)** Morphological analysis of microglia in the hippocampus (*n* = 3; **A**: sham+LP 4 h vs. VGX+LP 4 h, *p* = 0.5852; sham+LP 24 h vs. VGX+LP 24 h, *p* = 0.0423. **B**: sham+LP 4 h vs. VGX+LP 4 h, *p* = 0.7433; sham+LP 24 h vs. VGX+LP 24 h, *p* = 0.0430, Three-way ANOVA). **(C,D)** Morphological analysis of microglia in the hypothalamus (*n* = 3; **C**: sham+LP 4 h vs. VGX+LP 4 h, *p* = 0.8475; sham+LP 24 h vs. VGX+LP 24 h, *p* = 0.3821. **D**: sham+LP 4 h vs. VGX+LP 4 h, *p* = 0.5444; sham+LP 24 h vs. VGX+LP 24 h, *p* = 0.7709, Three-way ANOVA). **(E,F)** No difference in the increases in serum levels of IL-6 and IL-1β were observed between sham+LP group and VGX+LP group at 4 h and 24 h after lapartomy, indicating that acute vagotomy had no significant effect on peripheral inflammation within 24 h (*n* = 4; **E**: sham+LP 4 h vs. VGX+LP 4 h, *p* = 0.9882; sham+LP 24 h vs. VGX+LP 24 h, *p* = 0.7346. **F**: sham+LP 4 h vs. VGX+LP 4 h, *p* = 0.9988; sham+LP 24 h vs. VGX+LP 24 h, *p* = 0.7426, Three-way ANOVA).

Supplementary Figure 2 **(A–D)** Morphological analysis of microglia in the hippocampus and hypothalamus of WT mice (A: WT-Glu 4 h vs. WT-Ro+Glu 4 h, *p* = 0.0435; WT-Glu 24 h vs. WT-Ro+Glu 24 h, *p* = 0.0356; **B**: WT-Glu 4 h vs. WT-Ro+Glu 4 h, *p* = 0.0204; WT-Glu 24 h vs. WT-Ro+Glu 24 h, *p* = 0.0106; **C**: WT-Glu 4 h vs. WT-Ro+Glu 4 h, *p* = 0.0484; WT-Glu 24 h vs. WT-Ro+Glu 24 h, *p* = 0.1718; **D**: WT-Glu 4 h vs. WT-Ro+Glu 4 h, *p* = 0.0243; WT-Glu 24 h vs. WT-Ro+Glu 24 h, *p* = 0.1361, Three-way ANOVA). **(E–H)** Morphological analysis of microglia in the hippocampus and hypothalamus of Kit^W-sh^ mice (**E**: WT-Glu 4 h vs. Kit^W-sh^-Glu 4 h, *p* = 0.0400; WT-Glu 24 h vs. Kit^W-sh^-Glu 24 h, *p* = 0.0300; **F**: WT-Glu 4 h vs. Kit^W-sh^-Glu 4 h, *p* = 0.0221; WT-Glu 24 h vs. Kit^W-sh^-Glu 24 h, *p* = 0.0373; **G**: WT-Glu 4 h vs. Kit^W-sh^-Glu 4 h, *p* = 0.0200; WT-Glu 24 h vs. Kit^W-sh^-Glu 24 h, *p* = 0.0400; **H**: WT-Glu 4 h vs. Kit^W-sh^-Glu 4 h, *p* = 0.0491; WT-Glu 24 h vs. Kit^W-sh^-Glu 24 h, *p* = 0.0465, Three-way ANOVA).

Supplementary Figure 3 **(A)** Protein expressions of NR2B, PSD-95, Occludin and Tryptase in the hippocampus of mice were examined by Western blotting (*n* = 3; NR2B: WT-Glu 4 h vs. WT-Ro+Glu 4 h, *p* < 0.001; WT-Glu 24 h vs. WT-Ro+Glu 24 h, *p* = 0.9600. PSD-95: WT-Glu 4 h vs. WT-Ro+Glu 4 h, *p* = 0.9991; WT-Glu 24 h vs. WT-Ro+Glu 24 h, *p* = 0.0200. Occludin: WT-Glu 4 h vs. WT-Ro+Glu 4 h, *p* = 0.9998; WT-Glu 24 h vs. WT-Ro+Glu 24 h, *p* = 0.3300. Tryptase: WT-Glu 4 h vs. WT-Ro+Glu 4 h, *p* < 0.001; WT-Glu 24 h vs. WT-Ro+Glu 24 h, *p* = 0.9800, Three-way ANOVA). **(B)** The mRNA level of NR2B in the hypothalamus (*n* = 4; WT-Glu 4 h vs. WT-Ro+Glu 4 h, *p* = 0.0200; WT-Glu 24 h vs. WT-Ro+Glu 24 h, *p* = 0.9995, Three-way ANOVA). **(C)** Q-PCR analysis of the relative expression of NR2B mRNA in BMMCs (*n* = 4; con vs. Glu 4 h, *p* = 0.0050; Glu 4 h vs. Ro+Glu 4 h, *p* = 0.0300, One-way ANOVA).

## Abbreviations

POCD: postoperative cognitive dysfunction
CNS: central nervous system
yBBB: blood-brain barrier
LPS: lipopolysaccharide
MCs: Mast cells
BMCs: brain mast cells
NMDA: N-methyl-D-aspartate
NR2B: N-methyl-D-aspartate receptor subunit 2B
Ro: Ro25-6981
P-to-C: peripheral-to-central
WT: wild-type
C57BL/6-Kit^W-sh^/Kit^W-sh^: Kit^W-sh^
IACUC: Institutional Animal Care and Use Committee
LP: Laparotomy
VGX: Cervical vagotomy
BMMCs: bone marrow-derived MCs
PFA: paraformaldehyde
BSA: Bovine Serum Albumin
PBS: Phosphate-buffered saline
TFC: trace fear conditioning
qPCR: Quantitative real-time polymerase chain reaction
TBST: Tris buffered saline containing Tween 20
GO: Gene Ontology
KEGG: Kyoto Encyclopedia of Genes and Genomes
BP: biological process
CC: cellular component
MF: molecular function
PPI: Protein-protein interaction
MCC: maximal clique centrality
IL-6: Interleukin-6
IL-1β: Interleukin-1beta
TNF-α: tumor necrosis factor-alpha
TBI: traumatic brain injury
MS: multiple sclerosis
EAE: experimental autoimmune encephalomyelitis
AD: Alzheimer’s disease
NTS: nucleus tractus solitarius.
