# Supplementary figures and images for: Laparotomy-Induced Peripheral Inflammation Activates NR2B Receptors on the Brain Mast Cells and Results in Neuroinflammation in a Vagus Nerve-Dependent Manner
Source: Front Cell Neurosci. 2022 Feb 10;16:771156. doi: 10.3389/fncel.2022.771156 (PMC8866729; doi:10.3389/fncel.2022.771156)

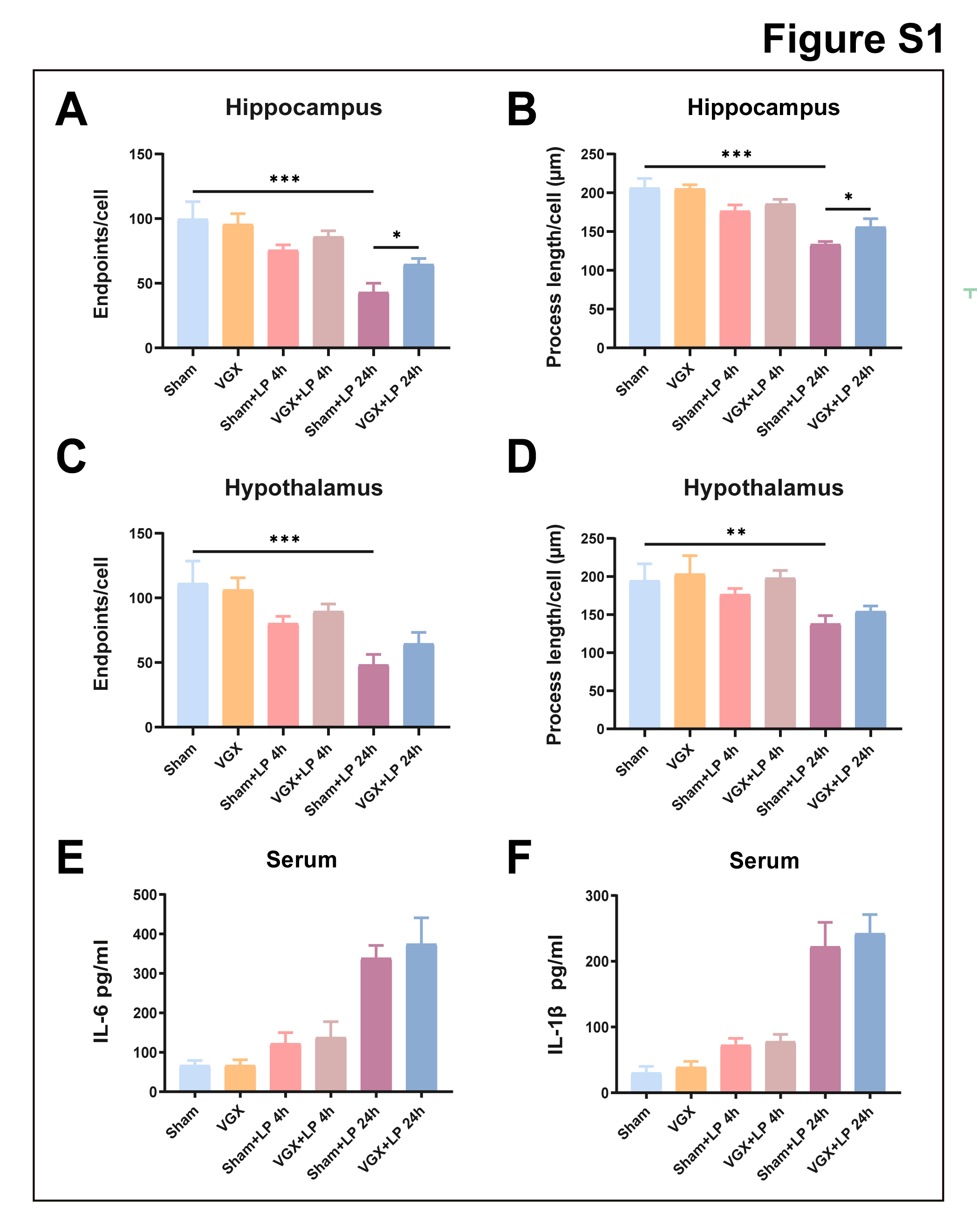

Supplement: Supplementary Figure 1 — (A,B) Morphological analysis of microglia in the hippocampus (n = 3; A: sham+LP 4 h vs. VGX+LP 4 h, p = 0.5852; sham+LP 24 h vs. VGX+LP 24 h, p = 0.0423. B: sham+LP 4 h vs. VGX+LP 4 h, p = 0.7433; sham+LP 24 h vs. VGX+LP 24 h, p = 0.0430, Three-way ANOVA). (C,D) Morphological analysis of microglia in the hypothalamus (n = 3; C: sham+LP 4 h vs. VGX+LP 4 h, p = 0.8475; sham+LP 24 h vs. VGX+LP 24 h, p = 0.3821. D: sham+LP 4 h vs. VGX+LP 4 h, p = 0.5444; sham+LP 24 h vs. VGX+LP 24 h, p = 0.7709, Three-way ANOVA). (E,F) No difference in the increases in serum levels of IL-6 and IL-1β were observed between sham+LP group and VGX+LP group at 4 h and 24 h after lapartomy, indicating that acute vagotomy had no significant effect on peripheral inflammation within 24 h (n = 4; E: sham+LP 4 h vs. VGX+LP 4 h, p = 0.9882; sham+LP 24 h vs. VGX+LP 24 h, p = 0.7346. F: sham+LP 4 h vs. VGX+LP 4 h, p = 0.9988; sham+LP 24 h vs. VGX+LP 24 h, p = 0.7426, Three-way ANOVA). [file Image_1.TIF]

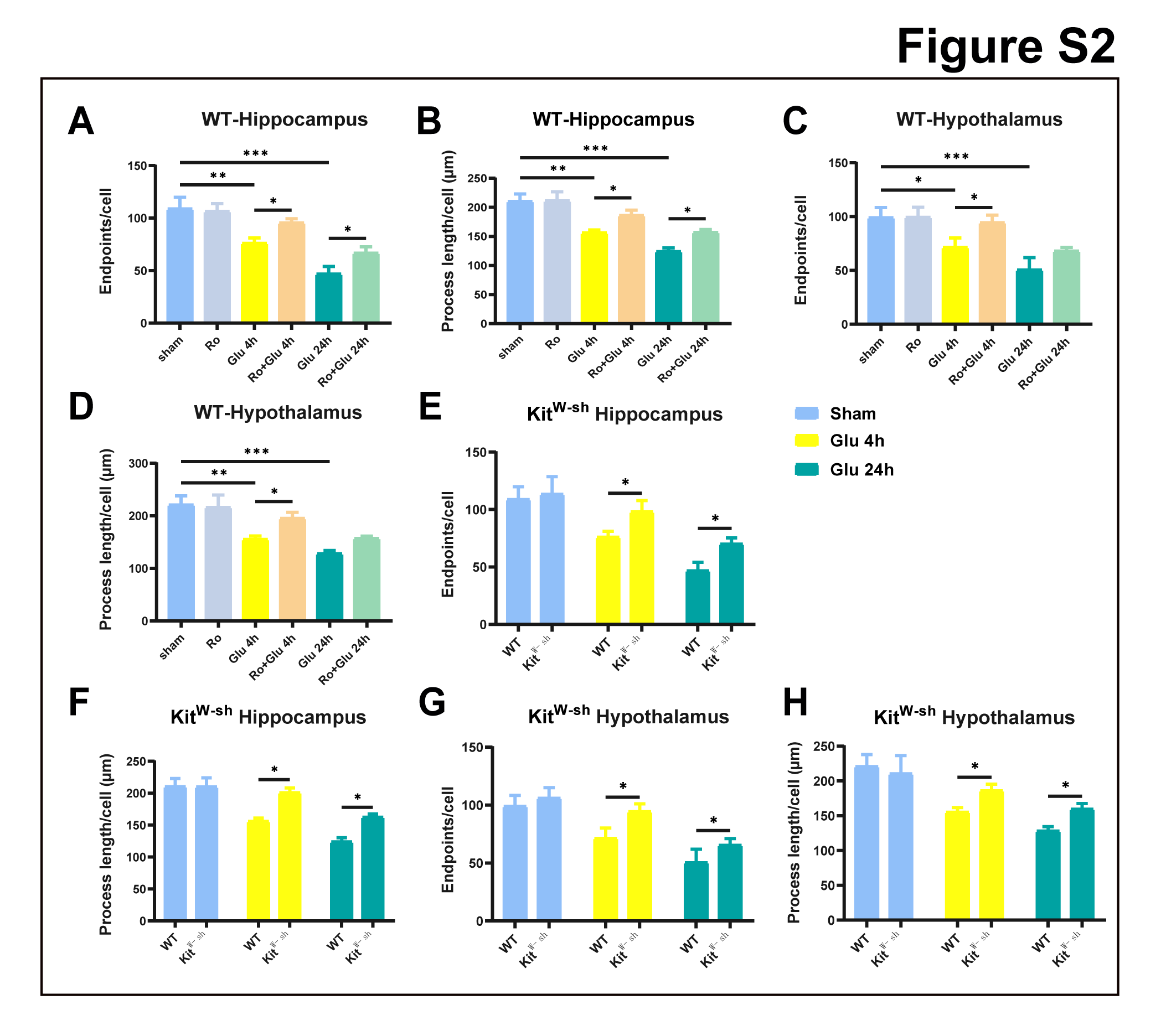

Supplement: Supplementary Figure 2 — (A–D) Morphological analysis of microglia in the hippocampus and hypothalamus of WT mice (A: WT-Glu 4 h vs. WT-Ro+Glu 4 h, p = 0.0435; WT-Glu 24 h vs. WT-Ro+Glu 24 h, p = 0.0356; B: WT-Glu 4 h vs. WT-Ro+Glu 4 h, p = 0.0204; WT-Glu 24 h vs. WT-Ro+Glu 24 h, p = 0.0106; C: WT-Glu 4 h vs. WT-Ro+Glu 4 h, p = 0.0484; WT-Glu 24 h vs. WT-Ro+Glu 24 h, p = 0.1718; D: WT-Glu 4 h vs. WT-Ro+Glu 4 h, p = 0.0243; WT-Glu 24 h vs. WT-Ro+Glu 24 h, p = 0.1361, Three-way ANOVA). (E–H) Morphological analysis of microglia in the hippocampus and hypothalamus of KitW-sh mice (E: WT-Glu 4 h vs. KitW-sh-Glu 4 h, p = 0.0400; WT-Glu 24 h vs. KitW-sh-Glu 24 h, p = 0.0300; F: WT-Glu 4 h vs. KitW-sh-Glu 4 h, p = 0.0221; WT-Glu 24 h vs. KitW-sh-Glu 24 h, p = 0.0373; G: WT-Glu 4 h vs. KitW-sh-Glu 4 h, p = 0.0200; WT-Glu 24 h vs. KitW-sh-Glu 24 h, p = 0.0400; H: WT-Glu 4 h vs. KitW-sh-Glu 4 h, p = 0.0491; WT-Glu 24 h vs. KitW-sh-Glu 24 h, p = 0.0465, Three-way ANOVA). [file Image_2.TIF]

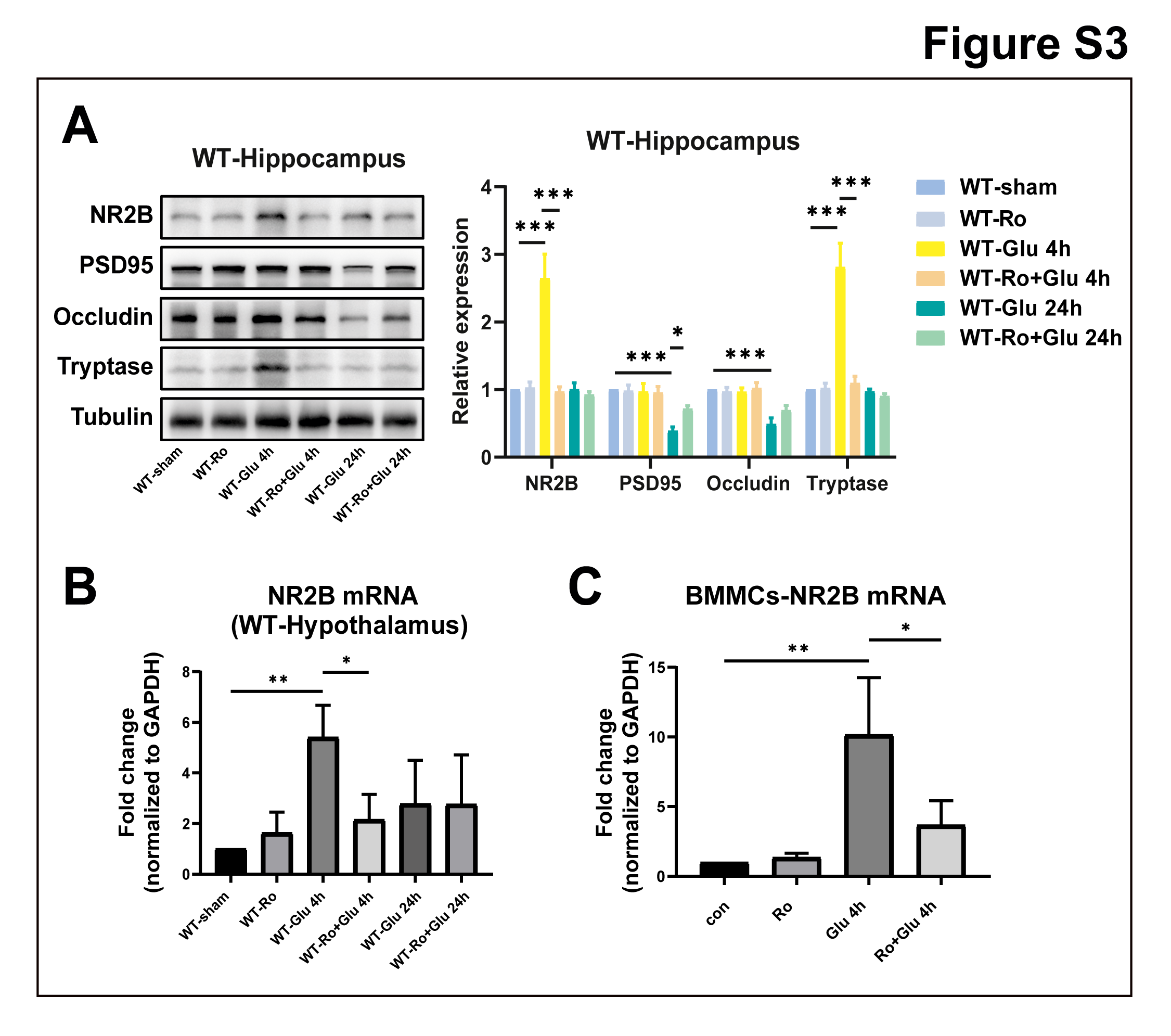

Supplement: Supplementary Figure 3 — (A) Protein expressions of NR2B, PSD-95, Occludin and Tryptase in the hippocampus of mice were examined by Western blotting (n = 3; NR2B: WT-Glu 4 h vs. WT-Ro+Glu 4 h, p < 0.001; WT-Glu 24 h vs. WT-Ro+Glu 24 h, p = 0.9600. PSD-95: WT-Glu 4 h vs. WT-Ro+Glu 4 h, p = 0.9991; WT-Glu 24 h vs. WT-Ro+Glu 24 h, p = 0.0200. Occludin: WT-Glu 4 h vs. WT-Ro+Glu 4 h, p = 0.9998; WT-Glu 24 h vs. WT-Ro+Glu 24 h, p = 0.3300. Tryptase: WT-Glu 4 h vs. WT-Ro+Glu 4 h, p < 0.001; WT-Glu 24 h vs. WT-Ro+Glu 24 h, p = 0.9800, Three-way ANOVA). (B) The mRNA level of NR2B in the hypothalamus (n = 4; WT-Glu 4 h vs. WT-Ro+Glu 4 h, p = 0.0200; WT-Glu 24 h vs. WT-Ro+Glu 24 h, p = 0.9995, Three-way ANOVA). (C) Q-PCR analysis of the relative expression of NR2B mRNA in BMMCs (n = 4; con vs. Glu 4 h, p = 0.0050; Glu 4 h vs. Ro+Glu 4 h, p = 0.0300, One-way ANOVA). [file Image_3.TIF]
